# Neutrophils in pregnancy: New insights into innate and adaptive immune regulation

**DOI:** 10.1111/imm.13392

**Published:** 2021-07-29

**Authors:** Serena Bert, Eleanor J. Ward, Suchita Nadkarni

**Affiliations:** ^1^ William Harvey Research Institute Barts and the London School of Medicine Queen Mary University London UK

**Keywords:** neutrophils, pregnancy, T cells

## Abstract

The immunology of pregnancy has been the focus of many studies to better understand how the mother is able to tolerate the presence of a semi‐allogeneic fetus. Far from the initial view of pregnancy as a state of immunosuppression, successful fetal development from implantation to birth is now known to be under the control of an intricate balance of immune cells. The balance between pro‐inflammatory functions used to promote embryo implantation and placental development and immunosuppressive activity to maintain maternal tolerance of the fetus is an immunological phenotype unique to pregnancy, which is dependent on the time of gestation. Neutrophils are one of a host of innate immune cells detected at the maternal–fetal interface, but very little is known of their function. In this review, we explore the emerging functions of neutrophils during pregnancy and their interactions with and regulation of T cells, a key adaptive immune cell population essential for the establishment of fetal–maternal tolerance.

## Graphical abstract

Neutrophils have often been seen as the ‘Bad Guys’ in pregnancy. However, increasing evidence suggests that these immune cells and their subtypes play an integral role in maintaining maternal–fetal tolerance. The aim of this review was to highlight the importance of neutrophils during pregnancy and the implications they have on the wider immune response in pregnancy.

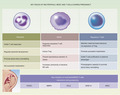

**Abbreviations**
ARG‐1: Arginase‐1
HLA: human leucocyte antigen
IFN‐γ: interferon gamma
IL: interleukin
LDNs: low‐density neutrophils
MDSC: myeloid‐derived suppressor cell
MHC‐II: Major Histocompatibility Complex class II
NETs: neutrophil extracellular traps
NK: Natural killer cell
PMN: polymorphonuclear neutrophil
ROS: reactive oxygen species
TGF‐β: transforming growth factor beta
Th: T helper T cell
TNF‐β: tumour necrosis factor beta

## INTRODUCTION

The maternal immune system undergoes major adaptations during pregnancy, to protect both the mother and fetus from pathogenic insult while also maintaining tolerance to the fetal allograft. This is a complex immunological balancing act, which has been the focus of research since the seminal work by Billingham et al. [1] more than 50 years ago using pregnancy as an example of immune tolerance. Unsurprisingly, there has been a significant increase in our knowledge since these initial observations with an increasing focus on the individual roles of various components of the innate and adaptive immune system. In this review, we will discuss the individual functions of T cell and neutrophils in pregnancy and how the interactions of these two subsets contribute to supporting a normal healthy pregnancy.

## DYNAMICS OF THE IMMUNE SYSTEM AT THE MATERNAL–FETAL INTERFACE

The interface between the maternal decidua and embryonic‐derived trophoblasts is a dynamic microenvironment where multiple interactions occur between cells of fetal and maternal origin. Among the cells found in this environment, 30%–40% of maternal cells are leucocytes recruited to the maternal–fetal interface during gestation, including natural killer (NK) cells, dendritic cells (DCs), macrophages, neutrophils and T lymphocytes [2]. Maternal immune cells accumulate at this site in response to the foreign tissues of the fetus, where they play a key role in early events of pregnancy, including decidualization, trophoblast invasion and spiral artery remodelling, thereby maintaining normal placentation.

The initial stage of pregnancy is characterized by the attachment of the embryo to the receptive endometrium in a process known as implantation. This event anchors the embryo to the uterine epithelium and requires functional and morphological modification of the maternal endometrium to form the decidua. [3] This is followed by proliferation and migration of the outermost layer of the embryo, known as the trophoblast layer, which invade the maternal tissues, forming a cellular barrier between the fetus and the maternal blood. Together, the decidua and the trophoblast layer constitute the maternal and fetal‐derived components of the placenta, which allows the exchange of gas, nutrients and waste between the maternal blood and the developing fetus [3, 4]. During these early stages of pregnancy, innate immune cells constitute the majority of leucocytes at the maternal–fetal interface, with the most abundant populations being decidual natural killer (dNK) cells and macrophages, comprising ~70% and ~20% of first‐trimester decidual leucocytes, respectively [2]. These myeloid cell populations, along with dendritic cells and neutrophils, have been shown to be major contributors to correct development of the placental bed. They are involved in promoting trophoblast invasion via cytokine production, regulation of tissue remodelling and spiral artery modification through secretion of pro‐angiogenic factors [5, 6, 7, 8, 9, 10].

Lymphocytes are another key immune cell population, with T cells in particular constituting 10%–20% of decidual leucocytes in the first trimester of gestation [2]. During pregnancy, the presence of the semi‐allogeneic fetus leads to the development of lymphocytes specific for fetal antigens, including paternal HLA‐C expressed on fetal trophoblasts [11] and fetal minor histocompatibility antigens (mHags) [12]. Antigen‐specific CD8+ T cells and activated CD4^+^CD25^dim^ T cells have been shown to expand both in the blood and in the decidua during pregnancy [13, 14, 15]. Antigen experienced CD8^+^ T cells from the blood of pregnant women maintain their cytotoxic potential [13]. To avoid reactivity against the fetus, a number of immunoregulatory mechanism come into play to carefully control the maternal adaptive immune system's effector functions and to establish fetal tolerance. These include dampening of CD8^+^ T‐cell cytotoxicity at the maternal–fetal interface [15] and shift towards a Th2 immunity for large part of gestation and increased recruitment and expansion of regulatory T cells [14, 16, 17]. The suppression of both fetus‐specific and non‐specific immune responses is particularly a fundamental event for successful gestation in which regulatory T cells and their interactions with other immune cells play a central role [17, 18, 19, 20].

Although it has been established that immune cells play a fundamental role in successful pregnancy outcome and their alterations can lead to complications in gestation, the complete picture of all the different regulatory mechanism, the cross‐talk between both immune cells and non‐immune cells and the variations in phenotype brought by the microenvironment at the maternal–fetal interface is largely unknown.

## THE ROLE OF T CELLS IN MODULATING TOLERANCE OF THE SEMI‐ALLOGENEIC FETUS

T lymphocytes are among the immune cells recruited to the maternal–fetal interface during gestation, with CD4^+^ T helper cells and CD8^+^ cytotoxic T cells comprising 35%–45% and 45%–75% of total T lymphocytes in the human decidua [2]. As these cells are able to recognize fetal antigens, it was initially proposed that maternal adaptive immunity is kept suppressed during pregnancy in order to avoid alloreactive immune responses against the fetus [21]. This has led to the portrayal of pregnancy as a Th2 predominant state to maintain immune tolerance, but more recent studies have indicated that regulation of T‐cell immunity during pregnancy is actually very dynamic and complex, with different T‐cell subsets contributing to successful gestation. It has been in fact proposed that three immunological phases can be distinguished in pregnancy: an initial pro‐inflammatory phase during implantation, trophoblast invasion and placentation, a subsequent anti‐inflammatory state during fetal growth and development, and lastly newly increased inflammation during labour [22]. The first‐trimester human decidua presents an increase in Th1 cells and regulatory T cells compared to the blood, while the percentage of decidual Th2 cells were shown to not differ from the blood [23]. The placental microenvironment at this stage requires pro‐inflammatory Th1 cytokines, among which IL‐6, IL‐1, IL‐15, IFN‐γ and IL‐8, which contribute to modest inflammation at the maternal–fetal interface and support embryo implantation, trophoblast invasion, tissue remodelling and recruitment of immune cells [24, 25, 26].

As an excessive and prolonged Th1 response can be adverse leading to the onset of pregnancy complications, an early event that is fundamental for establishment of fetal tolerance is the expansion of regulatory T cells (Treg) [27]. CD4^+^ CD25^+^ FoxP3^+^ Tregs have been shown to increase systemically during the first trimester of pregnancy in an antigen‐independent way [28], but an even more substantial increase in these cells can be observed in the uterus and draining lymph nodes [23, 29, 30]. The migration and expansion of regulatory T cells at the maternal interface are in fact an essential step in successful embryo implantation [31] that is promoted by both fetal‐derived alloantigens [23, 29] and by trophoblast and decidual‐derived factors, such as vasoactive intestinal peptide (VIP) [32]. The expansion of maternal Tregs is sustained throughout all three gestational stages and an increase in Treg even persists after parturition, although their proliferation is halted. This also favours Treg expansion in successive pregnancies [17]. Contributing to this increase is also induction of regulatory T cells during pregnancy by different innate immune cell populations, including neutrophils, myeloid‐derived suppressor cells and NK cells [18, 19, 20].

Decidual Tregs present a broad array of suppressor functions, which promote the establishment of immunological tolerance towards the fetus and are essential during gestation. They inhibit lymphocyte proliferation and response to paternal alloantigens, produce a number of immunosuppressive cytokines IL‐10 and TGF‐β and express surface markers CTLA‐4 and PD‐L1, involved in T‐cell inhibition [17, 18, 28, 29, 31]. Lastly, they can present pro‐angiogenic activity, favouring spiral artery remodelling [18, 31].

Although moderate inflammation is important in shaping the maternal–fetal interface during implantation and placental development, after this stage the establishment of a pro‐tolerogenic environment becomes fundamental in avoiding adverse immunological responses against the semi‐allogeneic fetus. One of the key features is a shift in the balance between Th1 and Th2 immunity and in their cytokine ratio.

Th1 cells are most known for their role in promoting cellular immunity through the release of pro‐inflammatory cytokines, such as IL‐2, IFN‐γ and TNF‐β, while Th2 cells are involved in humoral immunity and their cytokine profile includes IL‐4, IL‐6, IL‐13 and IL‐10.

For a significant proportion of gestation, the Th1/Th2 balance is tilted in favour of Th2 immunity, which favours dampening of Th1 immunity to promote immunological tolerance of the fetus [33, 34]. Cytokine profiles from PBMCs of pregnant women were shown to present an increase in Th2 cytokines IL‐4, IL‐10 and decrease in Th1 cytokines IFN‐γ and IL‐2 [35], and a bias towards Th2 cytokines was also detected in mouse pregnancy [36]. The importance of this balance in Th‐mediated immunity has been highlighted by the fact that pregnancy complications such as pre‐eclampsia and recurrent miscarriage present a bias to Th1 immunity and an increase in Th1/Th2 cytokine ratio [33, 35, 37, 38, 39]. This paradigm has now been expanded to include Th17 and Treg as well. Regulatory T cell‐mediated immune tolerance has been established as a key factor in successful pregnancy, while decrease and dysfunction of Tregs are observed in pregnancy complications [17, 28, 33, 40]. The increase in Th17 cells has also been suggested to drive inflammation during pre‐eclampsia and abortion [33, 40]. Overall, the balance in Th and Treg subsets that characterizes pregnancy and their association with complications of gestation highlights the importance of better understanding the regulatory mechanisms and cell interactions involved in maintaining T‐cell homeostasis during pregnancy.

## PHENOTYPIC AND FUNCTIONAL HETEROGENEITY OF NEUTROPHILS IN PREGNANCY

Neutrophils are innate immune cells, which are classically viewed as first responders to foreign organisms and tissue damage. Thought to be a quite homogeneous population with a prominent role in inflammation, neutrophil heterogeneity has been under investigation in recent years and has shown that these cells can display different functions and phenotypes in both health and disease [41]. Aside from their key inflammatory functions, which include phagocytosis, production of cytotoxic granules and release of neutrophil extracellular traps (NETs), neutrophils have also been shown to display immunoregulatory functions, especially in the context of their cross‐talk with adaptive immune cells [42]. Of note, neutrophils are capable of producing a broad array of cytokines, both pro‐ and anti‐ inflammatory, and immunoregulatory factors in both mice and humans [43]. They have been shown to produce chemokines involved in the recruitment of T‐cell subsets, including Th1 and Th17 cells [44], and can acquire antigen‐presenting capabilities, expressing costimulatory molecules MHC‐II, CD80 and CD86 and contributing to T‐cell priming [45, 46]. Neutrophils have also been shown to possess a number of immunosuppressive functions, which are shared with myeloid‐derived suppressor cells (MDSC; discussed below), including inhibition of T‐cell proliferation and activation by ARG‐1 and by ROS production [47]. Granulocyte MDSCs and another subpopulation of neutrophils called low‐density neutrophils (LDNs) share similarities in that they are both found in the low‐density fraction following density gradient centrifugation and share several surface markers including CD66b, CD15 and CD33 [48]. LDNs were first described in patients with systemic lupus erythematosus where they exhibited pro‐inflammatory functions [49]. LDNs are increased both in pregnant SLE patients and in healthy pregnant patients but have contrasting functions. LDNs from pregnant SLE patients are inflammatory, whereas LDNs from healthy pregnancies express high levels of arginase and therefore are presumed suppressive [49, 50]. The contrasting functions of seemingly phenotypically similar neutrophils have led to the description numerous neutrophil subsets within the literature. However, this is a highly contentious subject, with many arguing the different nomenclature used is misleading, as no clear biological differences are observed in these subsets. McKenna et al. [48] have recently highlighted the similarities between supposed neutrophil populations: they suggest neutrophil plasticity accounts for the differences observed and propose using long‐standing nomenclature describing maturity rather than separating to subpopulations.

In pregnancy, the role of neutrophils is largely elusive. Studies have in fact indicated that these cells play a role in both regulating correct placental development and fetal tolerance but can also contribute to pregnancy complications. Thus, careful regulation of the inflammatory functions of these cells and promotion of their immunoregulatory functions most likely plays a role in successful gestation.

An increase in leucocytes and in particular of granulocytes is observed in the circulation of pregnant women, accompanied by increased expression of activation markers CD11b, CD11a and CD54 [51] and an increase in neutrophil products in the blood [52]. However, dampening of different pro‐inflammatory functions of neutrophils during pregnancy has been reported in the literature, which might be a mechanism contributing to maternal tolerance of the fetus.

This includes a decrease in phagocytotic activity [53] and oxidative burst [54, 55] in circulating neutrophils from pregnant women. NET formation is another well‐known pro‐inflammatory function of neutrophils, which must be carefully controlled during gestation. Neutrophils acquire a pro‐NETotic state in pregnancy through contact with placental‐derived factors [56]. The hormonal milieu established during pregnancy then regulates neutrophil NETosis by establishing a balance between pro‐NETotic activity of oestrogens and NET antagonism by progesterone [57]. NET release is also shown to be dampened through interactions with trophoblast‐derived factors, thus avoiding placental inflammation [58]. In pre‐eclampsia, the increase in neutrophil activation and elevated release of syncytiotrophoblast microparticles from the placenta result in a noticeable increase in NET release at the maternal–fetal interface [56]. Excessive NETosis that characterizes this pregnancy complication hinders trophoblast migration and could be a contributor to defective placental development, scarce perfusion and increased inflammation [56, 59]. NETs are also well renowned for their pro‐inflammatory effects and have also been shown to induce endothelial damage [60]. The increase in NET release might be involved in the elevation in maternal cell‐free DNA detected in plasma of pre‐eclamptic women, which was shown to correlate to severity of this disorder [61]. Together, these inflammatory changes induced by NETs may play an important role in pathogenesis of pre‐eclampsia and placental dysfunction.

At the maternal–fetal interface, neutrophils can be detected in the decidua from the first trimester of pregnancy [25] and increase during the second trimester of human gestation, while in murine pregnancy they peak during spiral artery angiogenesis [9]. Their recruitment during the first trimester is mediated by chemoattractants, including GM‐CSF produced by uterine epithelial cells [62] and CXCL8 and GM‐CSF produced by myeloid cells [25]. Placental factors also increase CXCL8 production by T cells [63], suggesting T cells play a role in promoting the migration of neutrophils to the placenta. Decidual neutrophils are key players in tissue remodelling and placental vascularization during the first and second trimesters of gestation. Their productions of MMP9, ROS and HGF contribute to successful placentation and aid embryo implantation during the peri‐implantation period [64, 65]. They have also been shown to play a fundamental role in spiral artery remodelling. Other than localizing in proximity of these structures, they were shown to acquire a pro‐angiogenic phenotype, producing VEGF‐A, ARG‐1 and CCL2 and inducing formation of vascular networks [9]. This phenotype is also favoured by the release of trophoblast‐derived factors [59].

Studies conducted by our laboratory have also put in evidence the importance of the placental microenvironment and pregnancy hormones in shaping neutrophil phenotype and functions. We observed in fact that exposure to oestrogens and progesterone induces an anti‐inflammatory phenotype in human neutrophils, characterized by low expression of CD62L and CD11b, while increasing expression and localization to the membrane of Annexin A1 (AnxA1) [18, 66]. The acquisition of a quiescent and anti‐inflammatory polarization by these neutrophils is a crucial event in gestation. In fact, we first observed by using a mouse model of neutrophil depletion that the absence of neutrophils at key time‐points in placental development leads to defective placentation and reduced fetal growth, indicative of a pre‐eclampsia‐like phenotype. Subsequent investigation in women with pre‐eclampsia showed that, unlike in uncomplicated pregnancies, circulating neutrophils from these women displayed an activated phenotype, with low expression of AnxA1 and lost their ability induce regulatory T cells (discussed below) [18].

Interestingly, attenuated plasma levels of progesterone and oestrogens have been described in pre‐eclampsia and was also observed in our cohort, suggesting that maternal hormones might be involved in modulating acquisition of the correct neutrophil phenotype during gestation [18, 67].

Accumulating evidence suggests activated pro‐inflammatory neutrophils play a role in the pathology of pre‐eclampsia. Circulating neutrophils from pre‐eclamptic women have increased ROS production capability compared to normal pregnancy [54, 68] and present increased CD11b expression [18, 56, 69, 70]. As mentioned previously, neutrophils from pregnant women have been found to present increased NETosis, which is promoted by interaction with syncytiotrophoblast microparticles (STBM) from the placenta. This function is noticeably increased in pre‐eclampsia, resulting in elevated NET presence within the placental architecture [56]. Increased neutrophil activation and NET formation can interfere with trophoblast migration, which could be contributing to defective placental development observed in pre‐eclampsia [59]. Neutrophils may also influence the activity of other leucocytes at the maternal–fetal interface. For instance, NET release primes T cells by lowering their activation threshold and upregulating expression of activation markers CD69 and CD25 in vitro [71].

Overall, this suggests that shifts in neutrophil polarization may greatly influence the microenvironment at the maternal–fetal interface and play important roles in pregnancy and its associated complications.

## THE CROSS‐TALK BETWEEN NEUTROPHILS AND T CELLS

While the roles of individual immune cells in the development and maintenance of pregnancy have been extensively studied, the study of the interaction between immune cell subsets is still in its infancy. As mentioned previously, CD4^+^ T cells play a crucial role in the induction and maintenance of maternal–fetal immune tolerance, with a bias towards a Th2 phenotype [36]. The mechanisms regulating T‐cell function in pregnancy are yet to be fully elucidated; however, there is growing evidence suggesting the interaction of neutrophil/ neutrophil‐like cells with T cells may be important. In non‐pregnancy settings such as in infection and autoimmunity, it is now well established that neutrophils modulate innate and adaptive immune responses. Neutrophils and T cells are known to reciprocally influence their effector functions through chemokine/cytokine production, NET release or contact‐dependent mechanisms. Neutrophils have been shown to modulate, both positively and negatively, a variety of CD4^+^ subsets including Th1, Th2, Th17 and Tregs [46, 72, 73]. Recent work suggests the outcomes of the neutrophil–T cell interactions are dependent on the activation status of both cell types [74]. Neutrophils populate the spleen and lymph nodes under homeostatic and inflammatory conditions and rapidly migrate from sites of infection to draining lymph nodes where they enhance T‐cell proliferation [75]. These neutrophils express molecules typically associated with antigen presentation functions, high levels of MHC class II and the costimulatory molecules CD80 and CD86. Abi Abdallah et al. [46] showed that neutrophils behave like professional APCs, processing ovalbumin (Ova) and presenting peptide antigen to Ova‐specific T cells and inducing the differentiation of both Th1 and Th17 cells. A neutrophil‐dendritic cell hybrid population that exhibits dual properties of both neutrophils and dendritic cells has been shown to differentiate when both immature and mature bone marrow‐derived neutrophils are cultured with granulocyte macrophage‐colony‐stimulating factor. This hybrid population expresses markers of both neutrophils (Ly6G) and DCs (CD11c, MHC‐II, CD80 and CD86) and exhibits properties reserved for these individual cell types such as probing motion, antigen presentation and extrusion of neutrophil extracellular traps and bactericidal properties via cathelicidin production [76, 77]. Importantly, neutrophils not only function as APCs for CD4^+^ T cells but also present antigen to CD8^+^ T cells [45, 78]. The role of neutrophil‐derived cathelicidin in regulating T‐cell responses is further demonstrated in a recent paper by Minns et al. [72]. Here, the authors demonstrated that neutrophil cathelicidin is important in controlling T‐cell responses by inducing Th17 cells, suppressing Th1 differentiation and inhibiting the death of IL‐17‐producing cells. Cathelicidin also plays a role in mediating pro‐inflammatory responses during labour [79]. In addition to cathelicidin, many other mediators such as MPO, elastase, lactoferrin, ARG‐1 and gelatinase are released from granules during neutrophil degranulation and have been demonstrated to affect the development of T‐cell responses [80]. Often, the effect of the granule contents on T‐cell responses are context‐dependent; MPO induces pro‐inflammatory T‐cell responses in patients with anti‐MPO glomerulonephritis due to its role as an autoantigen [81]. However, MPO can also suppress DCs and CD4+ T cells in vitro and in vivo [82]. Neutrophil elastase displays broadly anti‐inflammatory roles, supressing CXCL12 induced T‐cell transendothelial migration without affecting further neutrophil recruitment [83]. However, there have been a small number of reports suggesting a pro‐inflammatory role of elastase; the discovery of neutrophil elastase‐specific T cells and the requirement of elastase‐dependent cleavage of CXCL8 to generate Th17 responses [84, 85]. Enhanced arginase activity in placental neutrophils has been identified as one of the mechanisms used to maintain maternal–fetal tolerance during pregnancy. This arginase‐mediated T‐cell hyporesponsiveness also extends from the mother to the newborn with increased expression of arginase 1 in neonatal PMN being attributed to contributing to the impaired immune responses observed in neonates [86]. Further investigation into the role of neutrophil granule contents in controlling T‐cell responses in pregnancy is warranted. Another mechanism utilized by neutrophils to promote T‐cell activation is the release of neutrophil extracellular traps (NETs) [45, 78]. NETting neutrophils were in fact shown to promote priming of CD4^+^ T cells, increasing their expression of CD25 and CD69 and lowering their activation threshold. T‐cell activation by NETs required presence of dendritic cells (DCs), but were able to activate even when cultured with resting DCs [71].

Lastly, the cross‐talk between neutrophils and T cells involves their reciprocal recruitment in the setting of inflammation. LPS‐ and IFNγ‐stimulated neutrophils are capable of inducing Th1 and Th17 migration via production of chemokines CCL2, CXCL10, and CCL20, but not of Th2. On the other hand, activated Th17 cells can produce CXCL8, which promotes neutrophil recruitment to sites of inflammation. Th17 can also modulate neutrophil activation, increasing expression of CD66b and CD11b, through production of TNF‐α, IFN‐γ and GM‐CSF [44].

In addition to their role in stimulating T‐cell responses, there is significant evidence showing neutrophils can directly suppress T‐cell responses. Arginase 1 is released by neutrophil granules and functions to convert arginine to L‐ornithine. Arginine metabolism mediates T‐cell suppression via downregulation of the ζ‐chain in the T‐cell receptor complex and subsequent arrest of T‐cell proliferation [87]. Neutrophil‐derived ROS, such as hydrogen peroxide (H_2_O_2_), is another frequently reported mediator of T‐cell suppression. H_2_O_2_ suppresses T‐cell proliferations in a variety of ways including inducing apoptosis, downregulating TCR ζ and decreasing NF‐κB activation. Additionally, oxidative stress alters actin dynamics through the oxidation of cofilin, which causes impairment of immune synapse formation and deficient T‐cell activation [88]. The suppression of T‐cell activation requires high concentrations of H_2_O_2_; however, neutrophils can utilize a mechanism of cell‐cell contact mediated by MAC‐1 to deliver ROS directly to the immune synapse [89]. An additional mechanism of T‐cell suppression by neutrophils, which relies on cell‐cell contact, is the induction of T‐cell apoptosis through the ligation of T‐cell PD‐1 by PD‐L1 expressed on neutrophils [90]. T‐cell activation is also inhibited by the release of serine proteases elastase and cathepsin G from neutrophil granules, which inactivate T cell‐stimulating cytokines IL‐2 and IL‐6 and catalyse the cleavage of both IL‐2 receptor and Il‐6 receptor [91].

While it is clear the interactions of neutrophils and T cells are important in disease states, work in the pregnancy field is still in its infancy. However, previous work conducted in our laboratory revealed that a population of quiescent neutrophils, generated by exposure to pregnancy hormones, play an important role in the maintenance of maternal–fetal tolerance and placentation, through the induction of a unique population of pro‐angiogenic regulatory‐like CD4^+^ T cells expressing GARP^+^CD127^lo^FOXP3^+^. The Annexin‐A1‐dependent transfer of FOXO1 from hormone treated neutrophils to T cells in apoptotic bodies is required for the induction of Tregs. Conversely, activated neutrophils from pre‐eclamptic pregnancies failed to induce regulatory‐like CD4^+^ T cells [18]. Work exploring neutrophil–T cell interactions during pregnancy is limited, so far, to this single study, owing in part, to the challenges of working with neutrophils. Until recently, in vivo studies were restricted due to the limitations of depletion strategies, namely antibody specificity and the requirement for multiple injections for long‐term depletion [92]. Genetic ablation using the Cre‐lox system is being increasingly used, but it is not without its problems due to the requirement of a cell specific promotor. Neutrophil depletion has been achieved using the human MRP8 (hMRP8) promoter. However, the caveat is that it also causes reductions in some monocyte/macrophage populations [92]. This promotor has also been used to generate an inducible model of neutrophil depletion using the diphtheria toxin receptor, but this again requires multiple diphtheria toxin injections to maintain depletion. In vitro study of neutrophils also presents challenges due to their short half‐life, absence of proliferation and sensitivity to environmental conditions. Neutrophil functional plasticity often depends upon local cues including the microenvironment unique to pregnancy. However, knowledge of how neutrophils can influence the function of other cell types from other disease settings can inform research into their function in pregnancy and represents an exciting area for further research.

Within the pregnancy field, there has been a focus in recent years on the interaction of a population of neutrophil‐like cells called myeloid‐derived suppressor cells (MDSCs) with T cells. MDSCs are myeloid progenitor cells that suppress the function of other immune cells. In humans they can be divided into two main subgroups: granulocytic MDSC (G‐MDSC) and monocytic MDSC (M‐MDSC). Both subsets express the common myeloid marker CD33, but lack expression of the human leucocyte antigen, D‐related (HLA‐DR). G‐MDSCs also express granulocytic lineage markers CD15 and/or CD66b, whereas M‐MDSCs express the monocytic antigen CD14 [93]. In mice, GR‐MDSCs are defined as CD11b^+^/Ly6G^+^/Ly6C^lo^ and MO‐MDSC as CD11b^+^/Ly6G^−^/Ly6C^hi^ cells [94]. G‐MDSCs can be distinguished from mature neutrophils by their lower density and their sedimentation with mononuclear cells following density gradient centrifugation. MDSCs are a normal part of haematopoiesis; however, they are dramatically expanded in pathological conditions such as cancer. The accumulation of MDSCs in pregnancy was first described by Mauti et al. [95], who observed that these cells inhibited NK cell activity, providing a possible mechanism to explain the enhanced metastasis seen during pregnancy.

Pregnancy is associated with an expansion of MDSCs in the peripheral blood [96]. It remains unclear whether MDSC abundance changes within the different pregnancy trimesters, however, the consensus is that peripheral blood MDSCs decrease following birth, suggesting the important role they play in maintaining maternal–fetal tolerance. In early pregnancy, it is believed that circulating neutrophils can acquire a MDSC phenotype upon migration to the maternal–fetal interface following stimulation with decidua‐derived GM‐CSF. Stimulation of CD15^+^ neutrophils with GM‐CSF activates STAT5 signalling, leading to the induction PD‐L2 expression which consequently is used by the neutrophil to restrain T‐cell proliferation though PD‐1 signalling [97]. CXCR2 has also been implicated with the recruitment of G‐MDSCs in decidual tissue and induces arginase I, an effector molecule used by MDSCs to regulate T‐cell activity [98]. HLA‐G, a non‐classical major histocompatibility complex I molecule highly expressed by trophoblasts and elevated in its soluble form in pregnant serum, promotes the accumulation of G‐MDSCs and an increase in their suppressive activity, through the engagement of its receptor ILT4 and subsequent activation of the STAT3 pathway, resulting in IDO expression in the myeloid cells [99]. Placental G‐MDSCs can also be activated by trophoblasts via CXCR4 and can more potently suppress T‐cell proliferation than G‐MDSCs derived from peripheral blood. Placental G‐MDSC express high levels of ROS and can polarize CD4+ T cells to a Th2 phenotype in a contact‐independent manner [100]. Another suggested way decidual G‐MDSC maintain tolerance during pregnancy is by inducing regulatory T cells. Activation of the TGF‐β/β‐catenin pathway in G‐MDSCs has been shown to induce FOXP3 expression in CD4^+ ^CD25^−^ T cells following the interaction of these two cell types [101].

Failure of expansion of MDSCs has been associated with spontaneous abortion in both murine models and humans [100, 102, 103]. Li et al. [103] reported patients experiencing unexplained recurrent pregnancy loss have reduced decidual G‐MDSCs and attributed this with excessive MDSC apoptosis with a decreased expression of decoy receptor 2 (DcR2), which sensitized these cells to TNF‐related apoptosis–induced ligand (TRAIL)‐mediated apoptosis. A role for hypoxia inducible factor 1α (Hif‐1α) in maintaining MDSC homeostasis has also been suggested, as animals with Hif‐1α‐deficient myeloid cells exhibit decreased accumulation of MDSC in the uterus, diminished MDSC suppressive function and increased MDSC apoptosis, all of which have been proposed to contribute to the increase in spontaneous abortions observed in these mice [102]. MSDCs may have a wider function than just curtailing T‐cell responses, as decreases in MDSCs observed in a murine model of spontaneous abortions have been associated with an increase in the cytotoxicity of decidual NK [104]. Pregnancy hormones are known play an important role in maintaining fetal–maternal tolerance. Therefore, it is not surprising that 17β‐oestradiol (E2) via Stat3 signalling induces the expansion and activation of M‐MDSCs [105]. A decline in both E2 and Progesterone serum concentrations in patients suffering from early miscarriage correlates with a decline in the frequency of G‐MDSCs and a skewing towards Th1 responses [106]. Furthermore, serum E2 levels positively correlate with proportion of M‐MDSC in peripheral blood of women undergoing IVF, where an increase in MDSC in peripheral blood was shown to be predictive of positive IVF outcome [107].

Interestingly, patients with pre‐eclampsia have a reduced frequency of G‐MDSCs but not M‐MDSCs in peripheral blood, when compared to normal pregnancies. This suppressed expansion of G‐MDSCs in pre‐eclamptic patients is associated with lower levels of serum Arg‐1, a mediator known to be used by G‐MDSCs to suppress T‐cell responses. However, this study failed to show any consequences of reduced G‐MDSC frequency and Arg‐1 levels on T‐cell activation or expansion in maternal peripheral blood cells [108]. In addition to their direct role in supressing T‐cell responses, G‐MDSCs from healthy pregnant women can also release exosomes containing the classical MDSC effector enzymes iNOS and Arginase 1. These exosomes have been shown to suppress T‐cell proliferation cause the polarization of Th cells towards a Th2 cytokine response and induce Treg generation [109].

**FIGURE 1:**
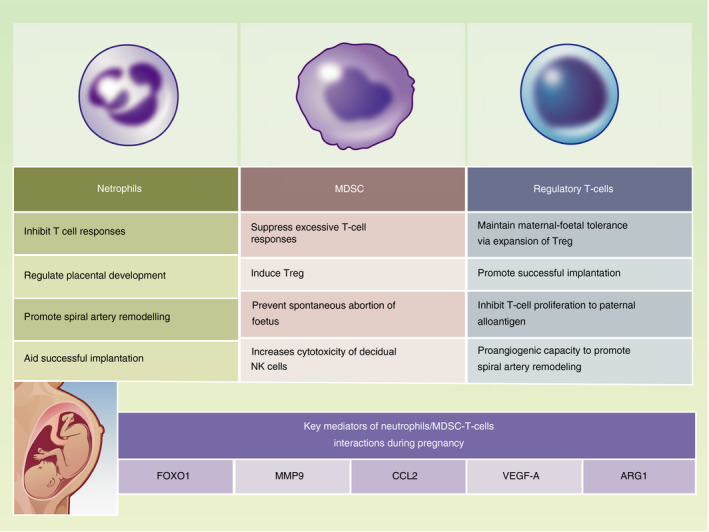
Key roles of neutrophils, MDSC and T‐cells during pregnancy

Not only do MDSCs play a role in modulating the immune response in gestation but there is also evidence to suggest MDSCs are important in the early neonatal period immediately after birth. In healthy adults, MDSCs are largely absent. However, there is an accumulation in both newborn mice and humans, with a gradual decline in their first weeks of life. G‐MDSCs, but not M‐MDSCs, are highly increased in cord blood when compared to peripheral blood of both adults and children and can effectively suppress T‐cell proliferation and cytokine production [110]. Further investigation has shown that G‐MDSCs isolated from cord blood inhibit Th1 responses in a contact‐dependent manner and induce Th2 responses and Tregs independent of contact via the expression of Arg‐1, ROS and inducible nitric oxide synthase, respectively [19]. Despite the increased risk of infection in preterm infants, Schwarz and colleagues [93] found G‐MDSCs are similarly increased in cord blood of term and preterm infants; however, if the infants were born from intraamniotic infection or suffered from postnatal sepsis, a further expansion of G‐MDSCs occurred. The expansion of G‐MDSCs in response to inflammatory insult further points towards their importance in regulating the neonatal immune response. Hu et al (2018) propose the presence of MSDCs is a mechanism to control the inflammatory response during microbial colonization of the neonate. They showed that lactoferrin, a protein found in the mother's milk, induces the upregulation of S100A9/A8 and *nos2* and nitric oxide; key molecules responsible for the suppressive activity of G‐MDSCs and M‐MDSCs, respectively [111]. Interestingly, G‐MDSCs, but not MDSCs, have been found in breast milk and suppress T‐cell proliferation and modulate Toll‐like receptor expression on monocytes. This finding further supports a role for MDSCs in regulating neonatal immune responses, particularly in the gut [112] (Figure 1).

## CONCLUDING REMARKS

The importance of T cells in the establishment and maintenance of pregnancy is widely known. However, the role neutrophils play in pregnancy have been less extensively studied. Much of the knowledge we have in relation to the wider functions of neutrophils, in addition to their antimicrobial actions, has been garnered from studies in infection, autoimmunity and cancer settings. However, emerging evidence suggests that neutrophils are important in all stages of pregnancy from implantation, placentation and associated tissue remodelling to birth. Further studies into the mechanisms involved in regulating neutrophil phenotype and activity during different steps of pregnancy, as well as their interactions with both immune and non‐immune cell populations of both of maternal and fetal origin, may provide insights into the breadth of action of these cells in gestation. Investigations exploring how changes in neutrophil phenotype during pregnancy complications might affect their interaction with different T‐cell subsets would also be insightful. Pro‐inflammatory cues from activated neutrophils could be involved in the increase in inflammation observed in pre‐eclampsia and other pregnancy‐associated complications. On the other hand, loss of neutrophil suppressive and immunoregulatory function might also be an important driver of these diseases. Of particular interest is the ability of neutrophils to recruit and or interact with T cells to modulate T‐cell function; a key event in the immunological balancing act that occurs during pregnancy. The interaction of a neutrophil‐like subset of cells called MDSCs with T cells has been a major focus within the field; however, further work is required to fully elucidate the full extent of these interactions as many of these studies have been observational. Further mechanistic studies investigating how neutrophils and T cells interact and the functional consequences of such interactions may provide useful insights into how alterations in the immune system contribute to pathological pregnancy, and lead to the development of interventions to treat these complications. Dissecting these mechanisms in more detail will undoubtedly increase our understanding of the role of neutrophils in pregnancy and could pave the way for novel therapeutic targets to treat pathological pregnancies such as pre‐eclampsia.

## CONFLICT OF INTERESTS

None.
